# Incidence of and Factors Associated With Nonfatal Self-injury After a Cancer Diagnosis in Ontario, Canada

**DOI:** 10.1001/jamanetworkopen.2021.26822

**Published:** 2021-09-24

**Authors:** Christopher W. Noel, Antoine Eskander, Rinku Sutradhar, Alyson Mahar, Simone N. Vigod, Elie Isenberg-Grzeda, James Bolton, Julie Deleemans, Wing C. Chan, Ravleen Vasdev, Victoria Zuk, Barbara Haas, Stephanie Mason, Natalie G. Coburn, Julie Hallet

**Affiliations:** 1 Temerty Faculty of Medicine, University of Toronto, Toronto, Ontario, Canada; 2 Dalla Lana School of Public Health, University of Toronto, Toronto, Canada; 3 ICES, Toronto, Ontario, Canada; 4 Clinical Evaluative Sciences, Sunnybrook Research Institute, Toronto, Ontario, Canada; 5 Faculty of Medicine, University of Manitoba, Winnipeg, Manitoba, Canada; 6 Cumming School of Medicine, University of Calgary, Calgary, Alberta, Canada

## Abstract

**Importance:**

Psychological distress is a key component of patient-centered cancer care. While a greater risk of suicide among patients with cancer has been reported, more frequent consequences of distress, including nonfatal self-injury (NFSI), remain unknown.

**Objective:**

To examine the risk of NFSI after a cancer diagnosis.

**Design, Setting, and Participants:**

This population-based retrospective cohort study used linked administrative databases to identify adults diagnosed with cancer between 2007 and 2019 in Ontario, Canada.

**Exposures:**

Demographic and clinical factors.

**Main Outcomes and Measures:**

Cumulative incidence of NFSI, defined as emergency department presentation of self-injury, was computed, accounting for the competing risk of death from all causes. Factors associated with NFSI were assessed using multivariable Fine and Gray models.

**Results:**

In total, 806 910 patients met inclusion criteria. The mean (SD) age was 65.7 (14.3) years, and 405 161 patients (50.2%) were men. Overall, 2482 (0.3%) had NFSI and 182 (<0.1%) died by suicide. The 5-year cumulative incidence of NFSI was 0.27% (95% CI, 0.25%-0.28%). After adjusting for key confounders, prior severe psychiatric illness, whether requiring inpatient care (subdistribution hazard ratio [sHR], 12.6; 95% CI, 10.5-15.2) or outpatient care (sHR, 7.5; 95% CI, 6.5-8.8), and prior self-injury (sHR, 6.6; 95% CI, 5.5-8.0) were associated with increased risk of NFSI. Young adults (age 18-39 years) had the highest NFSI rates relative to individuals aged 70 years or older (sHR, 5.4; 95% CI, 4.5-6.5). The magnitude of association between prior inpatient psychiatric illness and NFSI was greatest for young adults (sHR, 17.6; 95% CI, 12.0-25.8). Certain cancer subsites were also associated with increased risk, including head and neck cancer (sHR, 1.5; 95% CI, 1.2-1.9).

**Conclusions and Relevance:**

In this study, patients with cancer had a higher incidence of NFSI than suicide after diagnosis. Younger age, history of severe psychiatric illness, and prior self-injury were independently associated with risk of NFSI. These exposures appeared to act synergistically, placing young adults with a prior mental health history at the greatest risk of NFSI. These factors should be used to identify at-risk patients.

## Key Points

**Question:**

How common is nonfatal self-injury (NFSI) after a cancer diagnosis, and what are the associated risk factors?

**Findings:**

In this cohort study of 806 910 adults with cancer, 0.3% of the population had an NFSI event within 5 years of diagnosis. Age, history of severe psychiatric illness, and prior self-injury were the factors most strongly associated with NFSI.

**Meaning:**

These findings suggest that age, prior severe psychiatric illness, and prior self-injury could be used to identify patients with cancer at risk of NFSI events.

## Teaser

This cohort study examines the risk of nonfatal self-injury following a cancer diagnosis.

## Introduction

A diagnosis of cancer often brings significant emotional and psychological distress.^1,2,3,4,5,6^ Risk of death and the repercussions of therapy can lead to protracted periods of stress. For both patients with and survivors of cancer, identification and treatment of distress remains a challenge.^2,3,4,5,6^ This may stem from a limited understanding of cancer-related distress and how to identify patients with the greatest risk of harm.

Previous studies have established that suicide rates are higher among patients who have cancer compared with the general population, with specific risk factors including history of psychiatric illness, male gender, advanced disease, White race, and unmarried status.^6,7,8,9,10,11,12,13,14,15^ Suicide, however, is just one manifestation of psychological distress. The burden of psychological distress is magnified when considering other severe sequalae, such as nonfatal self-injury (NFSI).^16,17^ NFSI is a serious psychiatric outcome in and of itself, but it has not been examined in patients with cancer.^8,18,19^ An understanding of the risk of NFSI in cancer patients, including the level and timing of risk, is needed to develop better supportive care. In this population-based study, we examined the incidence of NFSI after a cancer diagnosis as well as associated risk factors.

## Methods

Linked administrative health care data sets were used to conduct a retrospective population-based cohort study of adults with a new cancer diagnosis. ICES is a prescribed entity under Ontario’s Personal Health Information Protection Act (PHIPA). Section 45 of PHIPA authorizes ICES to collect personal health information, without consent. The use of the data in this study was authorized under section 45 and approved by ICES’ Privacy and Legal Office. The study was approved by the Research Ethics Board at Sunnybrook Health Sciences Centre and reported following the Reporting of Studies Conducted Using Observational, Routinely Collected Health Data (RECORD)^20^ and the Strengthening the Reporting of Observational Studies in Epidemiology (STROBE)^21^ reporting guidelines.

### Data Sources

Administrative data sets were linked using unique encoded identifiers and analyzed at ICES. The Ontario population has universally accessible and publicly funded health care through the Ontario Health Insurance Plan (OHIP). The Ontario Cancer Registry (OCR) captures cancers diagnosed in Ontario since 1964 and has a greater than 95% capture of incident cancers.^22,23^ The Registered Persons Database (RPDB) contains demographic data on individuals covered under OHIP.^24^ The Ontario Marginalization Index quantifies the degree of marginalization across the province, defined as the proportion of adults aged 20 years or older without a high school diploma, families who are lone parent families, individuals receiving government transfer payments, individuals aged 15 years or older who are unemployed, individuals considered low income, and households living in dwellings that are in need of major repair.^25^ The Canadian Institute of Health Information Discharge Abstract Database contains information on hospitalizations. The National Ambulatory Care Reporting System is a database of outpatient visits to hospital and ambulatory care settings. The Ontario Mental Health Reporting System analyzes and reports on all Ontario residents receiving adult mental health services. Further information on the data sets is included in eTable 1 in Supplement 1.

### Study Cohort

Adults (aged ≥18 years) with a diagnosis of cancer between January 1, 2007, and March 31, 2019, were identified using *International Statistical Classification of Diseases and Related Health Problems, Tenth Revision *(*ICD-10*) O.3 codes (eTable 2 in Supplement 1) from the OCR. If more than 1 cancer diagnosis existed during the study window, the earliest date was selected.

### Outcome

The primary outcome of interest was any NFSI event after the index cancer diagnosis. An NFSI event was defined as an emergency department visit where a self-injury of intentional intent (*ICD-10-CA* codes, X61-X84) or undetermined intent (*ICD-10-CA* codes, Y10-Y19 and Y28) was present in any diagnostic field.^26,27,28,29^ The latter codes were included because prior research has demonstrated that these events often represent deliberate self-harm.^26^ In the *ICD-10*, self-injury includes physical injury as well as self-poisoning by medications, alcohol, or toxic chemicals. When the event resulted in death during the same index admission (ie, a fatal self-injury event or a death by suicide), this was not considered an NFSI event and was instead flagged for the analysis that treats death from any cause as a competing risk. Individuals were followed up until date of death, date of last clinical contact with the health care system, or March 31, 2020, whichever occurred first.

### Covariates

All variables were measured at time of cancer diagnosis. Age and sex were both obtained from the RPDB and treated as categorical variables. Rural living was determined by the rurality index of Ontario and dichotomized as urban or rural based on a threshold score of 40.^30^ Socioeconomic status was defined based on the material deprivation index and reported in quintiles.^31^ Comorbidity burden was measured using the Elixhauser algorithm and dichotomized, with 4 or greater indicating high comorbidity burden.^32,33^ Cancer stage was reported per the American Joint Committee on Cancer seventh edition.^34^ Severe psychiatric illness in the 5 years prior to cancer diagnosis was operationalized as a 4-level ordinal variable representing increasing levels of severity of mental health care needs.^13^ These levels were defined as follows: (1) inpatient severe psychiatric illness: any hospitalization for depressive, bipolar, or psychotic disorder; (2) outpatient severe psychiatric illness: 2 or more psychiatry outpatient visits or an emergency department visit for depressive, bipolar, or psychotic disorder; (3) other mental illness: fewer than 2 psychiatry outpatient visits with any physician (eg, psychiatrist or family physician) or emergency department visit with any mental illness diagnosis codes; and (4) none: no history of mental health services use.^13^ Self-injury in the 5 years prior to cancer diagnosis was treated as a dichotomous (ie, yes or no) variable. Further information can be found in eTable 3 in Supplement 1.

### Statistical Analysis

Baseline characteristics were examined using descriptive statistics for the entire cohort. NFSI was modeled as a time-to-event variable, measured from the date of cancer diagnosis. A cumulative incidence function was estimated to examine the risk of NFSI over time, in which death from any cause was incorporated as a competing risk.^35^ This analysis was conducted for the overall cohort, and then stratified by (1) history of severe psychiatric illness and (2) history of NFSI.

To explore the association between potential prognostic factors and NFSI, we constructed a series of multivariable Fine and Gray hazard models.^36,37^ As repeated NFSI events were found to be relatively rare (49 of 2482 patients [2.0%] in this study), this was not modeled as a recurrent event. Relevant demographic and clinical characteristics were identified a priori for inclusion in the model based on clinical relevance and the existing literature.^38,39^ The estimates from the multivariable model were further adjusted for year of diagnosis to account for potential secular trends in cancer care and survival. Collinearity was assessed, defined as variance inflation factor 2.5 or greater. We report subdistribution hazard ratios (sHRs) with 95% CIs.

We conducted 2 additional analyses. First, due to the known higher risk of psychiatric illness in younger adults and its potential association with the outcome, we added an interaction term between age and severe psychiatric illness history.^16^ Second, we added cancer stage into a second multivariable model to explore its potential association with outcomes. Cancer stage data were missing in 308 240 patients (38.2%) from our total cohort and missingness varied by cancer site (from 7.8% to 30.5% for breast, lung, colorectal, and prostate cancers to 99.8% for central nervous system cancers). We defined a subcohort of patients for whom missing cancer stage data was infrequent (breast, lung colorectal and prostate cancer) and included stage as an additional covariate.

All analyses were 2-sided, and statistical significance was set at *P* < .05. Analyses were conducted using SAS Enterprise Guide version 7.1 (SAS Institute) and R Studio version 12.1 (R Project for Statistical Computing).

## Results

In total, 806 910 patients met inclusion criteria (eFigure 1 in Supplement 1). The mean (SD) age was 65.7 (14.3) years; 405 161 patients (50.2%) were men; and the median (interquartile range [IQR]) duration of follow-up was 4 (2-8) years. The most common cancer sites included genitourinary (170 480 patients [21.1%]), gastrointestinal (152 092 [18.8%]), breast (112 943 [14.0%]), bronchopulmonary (103 079 [12.8%]), and hematopoietic and lymphoma (93 683 [11.6%]). A prior inpatient psychiatric history was documented in 6590 patients (0.8%) and prior self-injury in 2807 (0.3%). In those with prior self-injury, the median (IQR) time between the most recent self-injury event and cancer diagnosis was 33 (16-47) months. In total, 352 050 patients (43.6%) died during the study interval, including 182 (<0.1%) who died by suicide. Further demographic information of the cohort is detailed in Table 1.

**Table 1.** Characteristics of Included Patients

Characteristic	Patients, No. (%) (N = 806 910)
Sex	
Female	401 749 (49.8)
Male	405 161 (50.2)
Age, y	
18-39	38 414 (4.8)
40-49	65 108 (8.1)
50-59	146 691 (18.2)
60-69	216 709 (26.9)
≥70	339 988 (42.1)
Severe psychiatric illness history^a^	
Inpatient	6590 (0.8)
Outpatient	14 257 (1.8)
Other	296 112 (36.7)
None	489 951 (60.8)
Prior self-injury	2807 (0.3)
Material deprivation quintile	
1, least deprived	166 023 (20.6)
2	160 241 (19.9)
3	158 662 (19.7)
4	159 413 (19.8)
5, most deprived	160 089 (19.9)
Missing	2078 (0.3)
Residence	
Urban	724 827 (89.8)
Rural	81 538 (10.1)
Missing	545 (0.1)
High comorbidity burden (>4)	75 909 (9.4)
Primary cancer site	
Bronchopulmonary	103 079 (12.8)
Bone, sarcoma, and PNS	1659 (0.2)
Breast	112 943 (14.0)
CNS	10 669 (1.3)
Endocrine	32 335 (4.0)
Gastrointestinal	152 092 (18.8)
Genitourinary	170 480 (21.1)
Gynecologic	49 676 (6.2)
Hematopoietic and lymphoma	93 683 (11.6)
Head and neck	19 220 (2.4)
Skin	36 842 (4.6)
Other	24 232 (3.0)
Cancer stage at diagnosis	
0	1735 (0.2)
I	149 662 (18.5)
II	144 590 (17.9)
III	94 262 (11.7)
IV	108 191 (13.4)
Missing	308 470 (38.2)

Abbreviations: CNS, central nervous system; PNS, peripheral nervous system.

^a^ In 5 years prior to cancer diagnosis.

**Figure 1.**
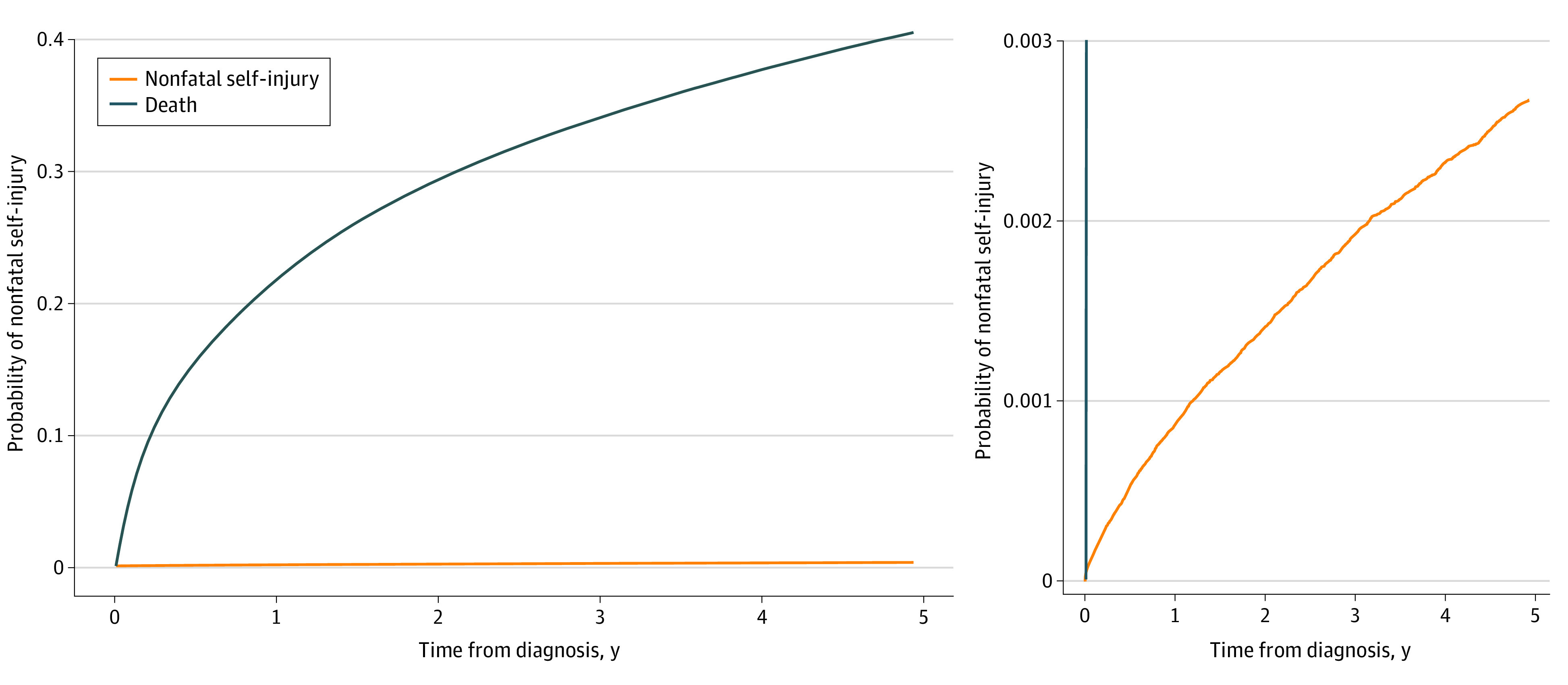
Cumulative Incidence Function of Nonfatal Self-injury After Cancer Diagnosis Accounting for the Competing Risk of Death From Any Cause The panel on the right is rescaled so that the self-injury cumulative incidence function can be better visualized.

**Figure 2.**
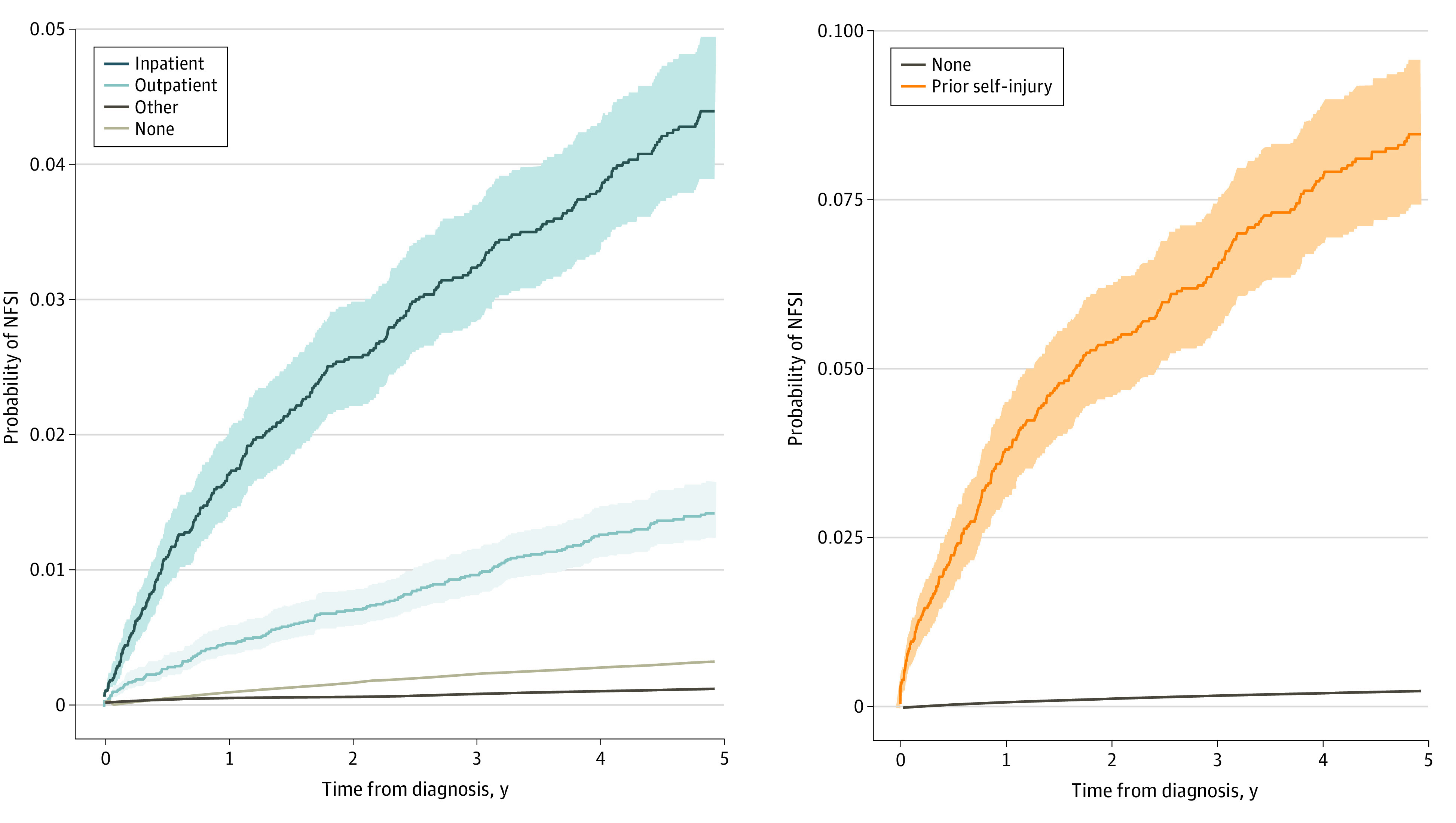
Cumulative Incidence Functions of Nonfatal Self-injury (NFSI) Stratified by Psychiatric Illness History and Prior Self-injury, Accounting for the Competing Risk of Death From Any Cause 95% CIs are represented by the shaded area.

There were 2482 NFSI events, including 49 patients (2.0%) who had a second NFSI event. Median (IQR) time from cancer diagnosis to NFSI was 29 (11-57) months (eFigure 2 in Supplement 1). Most NFSI events were related to medication poisoning (1924 [77.4%]) of intentional (1348 [54.3%]) or undetermined (576 [23.2%]) intent. The second most common reason was injury with a sharp object (263 [10.6%]). There were 10 individuals with an NFSI event who later went on to die by suicide during the follow-up period. The cumulative incidence of NFSI was 0.27% (95% CI, 0.25%-0.28%) at 5 years following cancer diagnosis (Figure 1). The 5-year cumulative incidence of NFSI was 10.2% (95% CI, 8.7%-11.7%) for patients with prior self-injury and 4.4% (95% CI, 3.9%-5.0%) for patients with a history of inpatient severe psychiatric illness (Figure 2).

Factors associated with NFSI in univariable and multivariable analyses are presented in Table 2. The strongest association with NFSI was found for a history of inpatient severe psychiatric illness (multivariable sHR, 12.6; 95% CI, 10.5-15.2), followed by outpatient severe psychiatric illness (multivariable sHR, 7.6; 95% CI, 6.5-8.8) and prior self-injury (multivariable sHR, 6.6; 95% CI, 5.5-8.0). Young adults (aged 18-39 years) were at the highest risk of NFSI relative to those aged 70 years or older (multivariable sHR, 5.0; 95% CI, 4.3-5.9). Additional factors associated with an increased risk of NFSI were higher material deprivation and greater comorbidity burden. Patients with head and neck cancers, genitourinary cancer, and hematologic cancer were at increased risk of NFSI relative to those with bronchopulmonary cancer. Rates of NFSI by specific cancer type are further outlined in Table 2.

**Table 2.** Factors Associated With Nonfatal Self-injury Events After Cancer Diagnosis, Fine-Gray Subdistribution Model

Characteristic	No.	Rate per 100 000	sHR (95% CI)	
			Univariable	Multivariable^a^
Sex				
Male	1153	66.3	1 [Reference]	1 [Reference]
Female	1329	72.4	1.2 (1.1-1.3)	1.0 (0.9-1.1)
Age, y				
18-39	307	134.5	5.5 (4.8-6.3)	5.0 (4.3-5.9)
40-49	464	120.6	4.7 (4.2-5.3)	4.1 (3.5-4.7)
50-59	644	82.7	2.9 (2.6-3.3)	2.6 (2.3-2.9)
60-69	561	53.8	1.7 (1.5-2.0)	1.7 (1.5-1.9)
≥70	506	44.4	1 [Reference]	1 [Reference]
Severe psychiatric illness history				
Inpatient	304	1270.8	34.1 (29.5-39.5)	12.6 (10.5-15.2)
Outpatient	214	362.9	10.7 (9.0-12.6)	7.6 (6.5-8.8)
Other	1181	89.8	2.5 (2.3-2.8)	2.5 (2.2-2.8)
None	783	36.0	1 [Reference]	1 [Reference]
Prior self-injury				
Yes	252	2452.9	33.9 (29.7-38.6)	6.6 (5.5-8.0)
No	2230	62.6	1 [Reference]	1 [Reference]
Material deprivation quintile				
1, least deprived	374	49.6	0.8 (0.7-0.9)	0.8 (0.7-1.0)
2	430	58.6	0.9 (0.8-1.1)	0.9 (0.8-1.1)
3	464	65.1	1 [Reference]	1 [Reference]
4	511	73.5	1.1 (1.0-1.2)	1.1 (1.0-1.2)
5, most deprived	666	101.5	1.4 (1.3-1.6)	1.2 (1.1-1.4)
Comorbidity burden				
Low, Elixhauser <4	2226	66.3	1 [Reference]	1 [Reference]
High, Elixhauser ≥4	256	118.7	1.1 (1.0-1.3)	1.2 (1.1-1.4)
Residence				
Urban	2219	68.8	1 [Reference]	1 [Reference]
Rural	263	76.2	1.1 (0.9-1.2)	1.1 (1.0-1.3)
Primary cancer site				
Bronchopulmonary	253	122.3	1 [Reference]	1 [Reference]
Bone, sarcoma, and PNS	<6	66.8	1.3 (0.5-3.0)	0.7 (0.3-1.8)
Breast	407	62.0	1.5 (1.3-1.7)	1.1 (0.96-1.4)
CNS	46	188.3	1.8 (1.3-2.4)	1.2 (0.9-1.6)
Endocrine	129	61.0	1.6 (1.3-2.0)	0.8 (0.7-1.1)
Gastrointestinal	367	69.2	1.0 (0.8-1.1)	1.0 (0.8-1.2)
Genitourinary	502	52.3	1.2 (1.0-1.4)	1.2 (1.1-1.5)
Gynecologic	176	72.0	1.5 (1.2-1.8)	1.1 (0.9-1.4)
Hematopoietic and lymphoma	312	83.9	1.4 (1.2-1.7)	1.3 (1.1-1.5)
Head and neck	93	108.8	2.0 (1.6-2.5)	1.5 (1.2-1.9)
Skin	109	54.6	1.4 (1.1-1.8)	1.1 (0.8-1.3)
Other	83	110.1	1.2 (0.98-1.5)	1.3 (0.98-1.3)

Abbreviations: CNS, central nervous system; sHR, subdistribution hazard ratio; PNS, peripheral nervous system.

^a^ Adjusted for diagnosis year.

**Figure 3.**
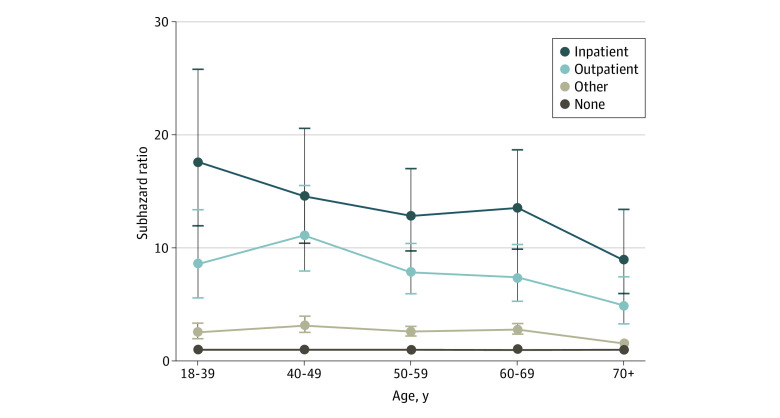
Interaction Effect of Age and Severe Psychiatric Illness History on the Risk of Nonfatal Self-injury Whiskers indicate 95% CIs (interaction term χ^2^ 39.7, *P* < .01).

An interaction term was added between age and severe psychiatric illness history and was significant. The magnitude of the association between severe psychiatric illness history and NFSI varied substantially by age (Figure 3). Young patients with an inpatient psychiatric history were at highest risk (sHR, 17.6; 95% CI, 12.0-25.8). Cancer stage was added to the multivariable model in the subcohort of patients with breast, bronchopulmonary, colorectal, or prostate cancers. Patients with advanced stage IV cancer had a lower risk of NFSI compared with stage I cancers (multivariable sHR, 0.5; 95% CI, 0.4-0.7). Adding stage to the model did not alter the findings regarding the association between other covariates and NFSI (eTable 4 in Supplement 1).

## Discussion

To our knowledge, this study is the first to document the incidence and risk factors for NFSI in patients with cancer. Overall, for every 1000 patients with cancer, 3 experience NFSI within 5 years of a cancer diagnosis, a much higher rate than that of suicide in this population. Age, history of severe psychiatric illness, and prior self-injury were the factors most strongly associated with the risk of NFSI. These risk factors interact, placing young adults with a history of severe psychiatric illness at the highest risk. Cancer factors associated with higher risk of NFSI were also identified, including the head and neck site and earlier-stage disease.

Cancer-related psychological distress can lead to significant repercussions for patients, including reduced understanding of the disease and adherence to therapy.^40,41,42,43^ The need for better management of cancer-related psychological distress has been emphasized by various cancer societies.^44,45^ While randomized trials have demonstrated the benefits of psychosocial interventions for patients with cancer and depressive symptoms, these same trials did not lead to a reduction in NFSI events.^46,47^ Accordingly, a systematic review on suicide and suicidal ideation in patients with cancer concluded there is a crucial need to define a high-risk cohort so that interventions for such groups could be further studied and developed.^48^

We identified that the risk of NFSI is higher in younger patients, those with severe psychiatric illness history, those with prior self-injury, those with cancer in specific sites, and those with earlier-stage disease.^16^ Many of the factors associated with NFSI were similar to those observed in the general population, although a few differ. Notably, female sex was not a risk factor for NFSI among patients with cancer.^16^ This may be related to different demographic characteristics in patients with cancer than in the general population. In the general population, the association between female sex and increased self-injury is seen mostly in younger age groups and reduces with advancing age.^49^ This cohort of cancer patients was older, which may explain the lack of association between sex and NFSI. The head and neck and genitourinary cancer sites were identified as being at highest risk of NFSI. This is in line with previous literature identifying patients with cancer in these disease sites as having particularly high risk of suicide.^10,11,13,50^ Possible explanations include potentially disfiguring treatments, high symptom burden, and shared risk factors between cancer development and mental health, such as alcohol use and smoking.^12,13,14,51,52,53,54,55,56,57^ The observation that patients with advanced cancers were less likely to engage in NFSI is interesting and unexpected. It may be that patients with advanced illness have more severe illness and have less opportunity for NFSI.

More than three-quarters of self-harm events were medication related, comparable with what is seen in the general population.^17^ While the intent and circumstances of NFSI cannot be ascertained from administrative health data, this could be associated with chemical coping. Chemical coping occurs when medications such as opiates or benzodiazepines are used to mask nonpain-related needs that are not being adequately addressed.^58,59,60^ While the drugs involved in the reported NFSI events are not detailed, opiates are among the most common drugs prescribed to patients with cancer. This finding and hypothesis require further investigation beyond the scope of the current study. Nonetheless, they highlight the importance of paying attention to patterns of prescribing and proactively addressing nonpain symptoms and distress.

Effective interventions for NFSI, such as cognitive behavioral therapy, exist and are supported by level 1 evidence.^61^ However, therapy should be targeted to underlying mechanisms. NFSI after a cancer diagnosis may be related to distress in different ways. NFSI is an extreme manifestation, beyond the typical distress associated with a cancer diagnosis. Patients with cancer who have poor coping mechanisms may relieve stress through self-injury without the intention of suicide, while others may become hopeless or suicidal. For NFSI associated with psychiatric disorders, treatment of the psychiatric disorder may reduce the risk, although NFSI associated with hopelessness may not be treatable in the same way. To implement tailored interventions, patients’ coping abilities and intentions need to be assessed.

Distress and depression have been recognized as significant burdens in patients with cancer, negatively impacting engagement, compliance, quality of life, and health care services utilization.^5,6,7,8^ Routine screening using validated instruments, rather than inconsistent clinicians’ judgment, has been recommended and implemented by cancer programs.^3,4,13,14,15^ Such screening programs, most often using patient-reported outcomes, could potentially support the identification of distress and risk of NFSI. However, the usefulness of screening is contingent on following up with psychosocial interventions.^43,62,63^ There is a gap between screening and interventions in clinical practice.^53,64,65,66,67,68^ For instance, the reporting of depressive symptoms is seldomly followed by intervention.^69^ Moreover, the association between screening patient-reported outcomes and manifestations of distress, such as NFSI, is unknown. The findings of this study have established NFSI as a measurable outcome that future work should address to further understand and tailor supportive care for patients with cancer. Future examination of the association between screening patient-reported outcomes and NFSI as well as cancer-specific screening tools for NFSI could support the identification of at-risk patients.

### Limitations and Strengths

This study has limitations. Several different phenotypes of NFSI exist, and a limitation of administrative data is that we are only able to capture the more severe type of NFSI that leads to a measurable interaction with the health care system. Consequently, our numbers likely underestimate the overall NFSI rate.^70^ There is also risk of differential misclassification bias. Deliberate self-injury may not be coded as such in hospital admission or death records, either through uncertainty about the motivation or a desire to spare the patient or family any perceived stigma.^26,71^ Nonetheless, this coding strategy has been used previously and shown to be highly specific.^26^ Moreover, we presume the date of diagnosis in the OCR is the date the patient became aware of the diagnoses; in reality, they could have been aware either before or after. Awareness of their cancer diagnosis prior to the date recorded in the OCR could threaten the validity of our findings. However, the median time between precancer self-injury and cancer diagnosis was approximately 3 years, indicating that most events occurred well before the cancer diagnosis. We also acknowledge that certain potential risk factors could not be assessed with administrative data, such as social network (including family and marital status), treatment-related factors, and environmental and genetic factors.^16^ Finally, medical assistance in dying (MAID) was introduced in Canada in 2016.^72^ While the availability of MAID may potentially alter patterns of NFSI, the legislation was rolled out in the last 3 years of our 13-year observation period, such that we do not anticipate it influenced our results. Despite these limitations, the strength of this study lies in its population-based design that allowed for a detailed and representative assessment of NFSI. We assembled the cohort, defined the covariates, and measured NFSI within a universal health system where access to care is not compounded by insurance status and loss to follow-up is minimal.

## Conclusions

Patients with cancer are at risk of NFSI. Young adults, patients with a history of severe psychiatric illness or prior self-injury, and those with head and neck or genitourinary cancer types had the highest risk. Certain risk factors acted synergistically, placing young adults with a history of severe psychiatric illness at the greatest risk of NFSI after a cancer diagnosis. This information is important for helping to devise strategies to identify and support patients at risk of NFSI after a cancer diagnosis.

## Notes

Supplement 1. **eTable 1. **Data Sources **eTable 2. **Strategy for Cohort Creation **eTable 3. **Main Variable Definitions Using Ontario Health Care Databases **eTable 4**. Factors Associated With Nonfatal Self-injury Events After Cancer Diagnosis Including Cancer Stage Data in Cancer Types With Most Complete Stage Information, Fine-Gray Subdistribution Model Accounting **eFigure 1. **Flowchart of Cohort Creation **eFigure 2.** Distribution of Time From Date of Cancer Diagnosis to Nonfatal Self-injury Event

Supplement 2.
Enhanced Supportive Psycho-oncology Canadian Care (ESPOC) Group Members
