# Supplementary material for: Incidence of and Factors Associated With Nonfatal Self-injury After a Cancer Diagnosis in Ontario, Canada
Source: JAMA Netw Open. 2021 Sep 24;4(9):e2126822. doi: 10.1001/jamanetworkopen.2021.26822 (PMC8463938; doi:10.1001/jamanetworkopen.2021.26822)
Supplement: Supplement 1. — eTable 1. Data Sources eTable 2. Strategy for Cohort Creation eTable 3. Main Variable Definitions Using Ontario Health Care Databases eTable 4. Factors Associated With Nonfatal Self-injury Events After Cancer Diagnosis Including Cancer Stage Data in Cancer Types With Most Complete Stage Information, Fine-Gray Subdistribution Model Accounting eFigure 1. Flowchart of Cohort Creation eFigure 2. Distribution of Time From Date of Cancer Diagnosis to Nonfatal Self-injury Event [file jamanetwopen-e2126822-s001.pdf]

Supplemental Online Content

Noel CW, Eskander A, Sutradhar R, et al; the Enhanced Supportive Psycho-oncology Canadian Care (ESPOC) Group. Incidence of and factors associated with nonfatal self-injury after a cancer diagnosis in Ontario, Canada. *JAMA Netw Open.* 2021;4(9):e2126822. doi:10.1001/jamanetworkopen.2021.26822

eTable 1. Data Sources

eTable 2. Strategy for Cohort Creation

eTable 3. Main Variable Definitions Using Ontario Health Care Databases

eTable 4 . Factors Associated With Nonfatal Self-injury Events After Cancer Diagnosis Including Cancer Stage Data in Cancer Types With Most Complete Stage Information, Fine-Gray Subdistribution Model Accounting

eFigure 1. Flowchart of Cohort Creation

eFigure 2. Distribution of Time From Date of Cancer Diagnosis to Nonfatal Self-injury Event

This supplemental material has been provided by the authors to give readers additional information about their work.

© 2021 Noel CW et al. *JAMA Network Open.*

eTable 1. Data Sources

Database	Description
**Ontario Cancer Registry (OCR)**	The OCR is a passive, provincial registry all incident cancer diagnoses in Ontario. It includes 96% of cancer diagnoses in the province. Information included in the registry: cancer topography and morphology/histology, and details on diagnosis (e.g., types of contributing information to the diagnosis, dates).
**Activity Level Report (ALR) of Cancer Care Ontario**	Cancer Care Ontario maintains a database of cancers-specific services, including consultations, chemotherapy, and radiotherapy provided by regional cancer centres in the province. Because all radiotherapy is delivered at RCCs, it is a complete source of information. However, because not all cancer patients who receive surgery or chemotherapy visit an RCC, it cannot be used as a population data sources for those treatments.
**Registered Patient Database (RPDB)**	The RPDB is an ICES database derived from all administrative data sources and provides demographic data including age, patient residence, vital status, date of last contact with the healthcare system, and OHIP eligibility.
**Ontario Registrar General (ORG)**	The ORG contains gold standard vital status data for all Ontarians. According to the Vital Statistics Act, it is mandatory to register all deaths occurring in the province.
**Ontario Marginalization Index (ONMARG)**	The ON-MARG is a specialized database using Census data to profile relative area-level marginalization dependency, deprivation, ethnic concentration, and instability at various geographic levels in Ontario.
**Ontario Health Insurance Plan (OHIP)**	The OHIP database contains all physician billing data including information on diagnoses as well as services provided, such as receipt of surgery, chemotherapy, and radiotherapy.
**National Ambulatory Care Reporting System (NACRS)**	The NACRS collects data elements describing emergency health services provided by EDs in Ontario, including diagnoses, procedures, and administrative information such as wait times.
**Ontario Drug Benefit Database (ODB)**	The ODB contains all information for prescription drug dispensed by community pharmacies and long-term care/nursing facilities. It covers all seniors (≥65 years old) and individuals on social assistance for all prescriptions listed on the provincial formulary.
**Ontario Mental Health Reporting System (OMHRS)**	The OMHRS collects information from adult psychiatric hospital admissions in Ontario.

© 2021 Noel CW et al. *JAMA Network Open.*

eTable 2. Strategy for Cohort Creation		
**Cancer site**		**ICD.O-3 code**
Bronchopulmonary:		C30.0 to 34.9, C38.1 to 39.9
Bone, Sarcoma, and PNS:		C47
Breast:		C50.0 to 50.9
CNS:		C71, C72, C70
Endocrine:		C73, C74, C75
Gastro-intestinal:		C15.0-15.9, C16.0-C16.9, C22.0, C22.1, C23.9, C24.0, C24.1, C24.8, C24.9, C25.0-25.9, C17.0-17.9, C18.0-C18.9, C21
Genito-urinary:		C64.9, C65.9, C66.9, C67, C68, C67.0-C67.9, C61, C74.0, C74.1, C74.9, C60, C62
Gynecologic:		C52.9, C53.0, C53.1, C53.8, C53.9, C54.0, C54.1, C54.2, C54.3, C54.8, C54.9, C55.9, C56.9, C57.0, C57.1, C57.2, C57.3, C57.4, C57.7, C57.8, C57.9, C51.0, 51.1, C51.2, C51.8, C51.9
Haematopoietic and lymphoma:		C77, C0 to C80 with histology code 959* to 972*
Head and Neck:		C10.0, C10.1, C10.2, C10.3, C10.4, C10.8, C10.9, C01.9, C02.0, C02.1, C02.2, C02.3, C02.4, C02.8, C02.9, C03.0, C03.1, C03.9, C04.0, C04.1, C04.8, C04.9, C05.0, C05.1, C05.2, C05.8, C05.9, C06.0, C06.1, C06.2, C06.8, C06.9, C07.9, C08.0, C08.1, C08.8, C08.9, C09.0, C09.1, 09.8, C09.9, C11.0, C11.1, C11.2, C11.3, C11.8, C11.9, C12.9 , C14.0, C14.2, C14.8, C76.0, C06.9, C14.8, C32.0, C32.1, C32.3, C32.8, C32.9, C13.0, C13.1, C13.2, C13.8, C13.9, C00.0, C00.1, C00.2, C00.3, C00.4, C00.5, C00.6, C00.8, C00.9, C14.8, C44.0
Skin:		C44.2 to C 44.9

© 2021 Noel CW et al. *JAMA Network Open.*

eTable 3. Main Variable Definitions Using Ontario Health Care Databases

Variable	Data source	Code strategy
Age at cancer diagnosis	Ontario Cancer Registry (OCR)	Categorical: 18-39, 40-49, 50-59, 60-69, ≥70
Sex	Registered Person Database (RPDB)	- Male<br>- Female
Prior Self-Injury	NACRS	See outcome definition: yes, no
Material Deprivation	Canadian Census<br>RPDB<br>Ontario Marginalization index (ONMarg)	Measure of the inability for individuals or households to afford goods or activities typical in a society at a given time – captured in quintiles.
Comorbidity Burden	CIHI-Discharge abstract database (DAD)<br>CIHI – Same day surgery (SDS) OHIP	Elixhauser score based on health services use with a 24-month look-back window prior to the date of cancer diagnosis<br>- High: sum score ≥4<br>- Low: sum score <4
Rural Residence	RPDB	Rural index of Ontario<br>- Rural: ≥40<br>- Urban: <40
Severe Psychiatric Illness History	CIHI-DAD<br>Ontario Mental Health Reporting System (OMHRS)<br>Ontario Health Insurance Plane	<u>Inpatient</u>: any CIHI-DAD or OMHRS admission with eligible mental health diagnoses codes (see list below)<br><u>Outpatient</u>: ≥2 psychiatry outpatient visit OR ED visit with eligible mental health (see list below) diagnoses codes<br><u>Other mental health services</u>: <2 psychiatry visits or ED visit with any mental health diagnoses codes or any family doctor visit with any mental health diagnoses codes<br><u>None</u>: all others<br><br>OHIP (ICD-9): 295-298, 311<br>CIHI-DAD((ICD-10): F20-29; F30-39<br>OMHRS (DSM-IV): Mood disorder, Schizophrenia, other
Cancer Site	OCR	<u>See Supplemental Table 2</u>
Cancer Stage	OCR	<u>Cancer stage at diagnosis using TNM system according to 7th edition of the AJCC:</u><br>- I<br>- II<br>- III<br>- IV<br>- Missing/unknown

© 2021 Noel CW et al. *JAMA Network Open.*

eTable 4. Factors Associated With Nonfatal Self-injury Events After Cancer Diagnosis Including Cancer Stage Data in Cancer Types With Most Complete Stage Information, (Fine-Gray Subdistribution Model Accounting

**Variable**		**sHR***	**[95% CI]**
**Age category** *(ref=≥70 or older)*	18-39	4.51	[3.21-6.35]
	40-49	3.84	[3.04-4.85]
	50-59	2.66	[2.20-3.22]
	60-69	1.75	[1.45-2.10]
**Sex** *(ref=Male)*	Female	1.02	[0.84-1.23]
**Rural residence** *(ref=Urban)*	Rural	1.11	[0.89-1.37]
**Deprivation Quintile** *(ref=Q3)*	1 (most deprived)	0.86	[0.68-1.07]
	2	0.92	[0.74-1.14]
	4	1.15	[0.94-1.41]
	5 (least deprived)	1.31	[1.07-1.59]
**High comorbidity burden** (Elixhauser ≥4) *(ref=low (<4))*		1.63	[1.31-2.02]
**Severe Psychiatric Illness History** *(ref=none)*	Inpatient	21.38	[16.97-26.9]
	Outpatient	8.93	[7.04-11.4]
	Other	2.20	[1.89-2.56]
**Cancer site** *(ref=Broncho-pulmonary)*	Breast	0.83	[0.66-1.05]
	Colo-rectal and enteric	0.93	[0.76-1.13]
	Prostate	0.95	[0.75-1.20]
**Cancer stage** *(ref=I)*	0	0.62	[0.15-2.49]
	II	0.86	[0.72-1.03]
	III	0.91	[0.74-1.11]
	IV	0.61	[0.48-0.78]
	Missing	0.93	[0.74-1.17]

*Adjusted for year of diagnosis

© 2021 Noel CW et al. *JAMA Network Open.*

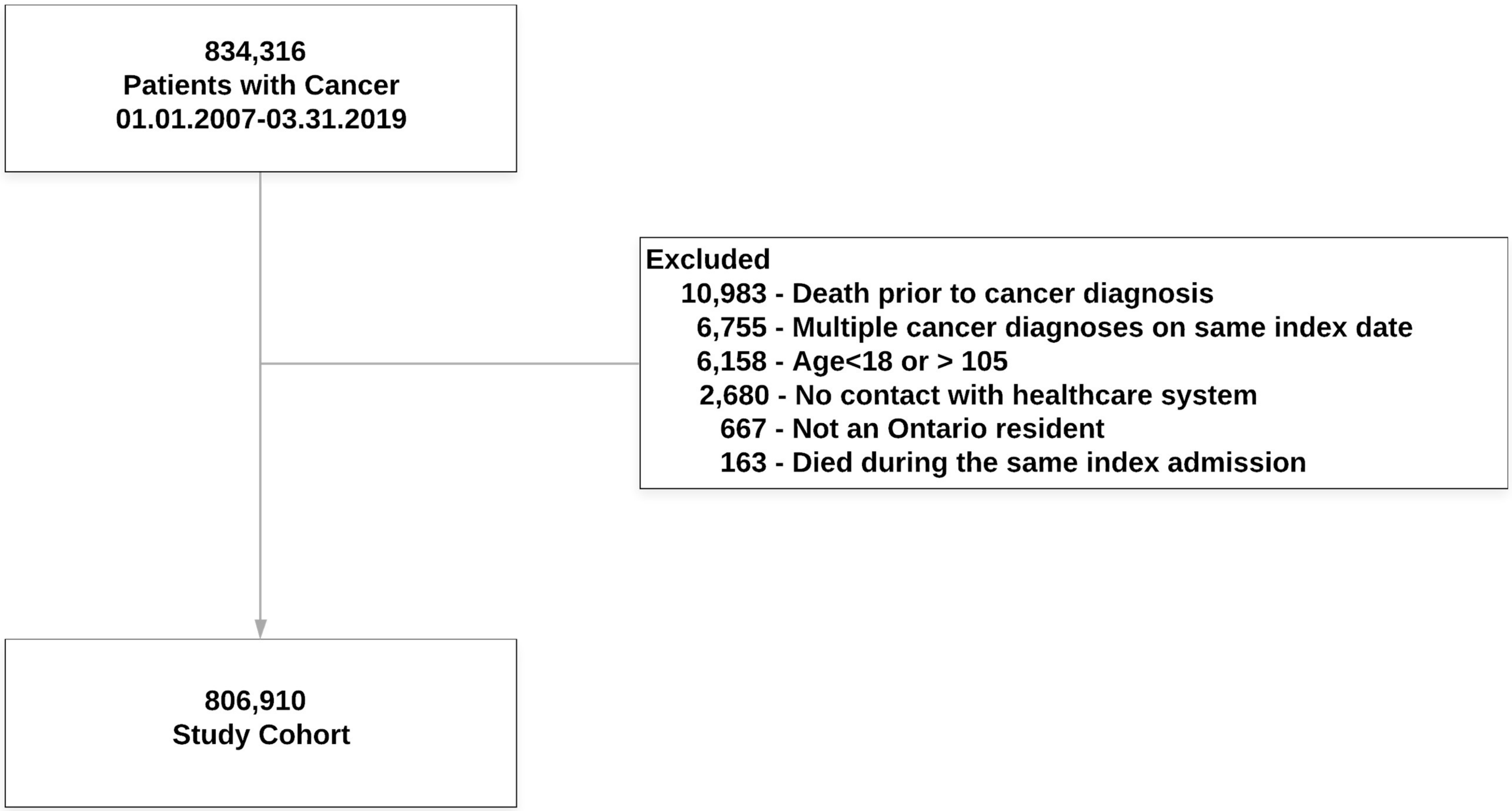

eFigure 1. Flowchart of Cohort Creation

© 2021 Noel CW et al. *JAMA Network Open.*

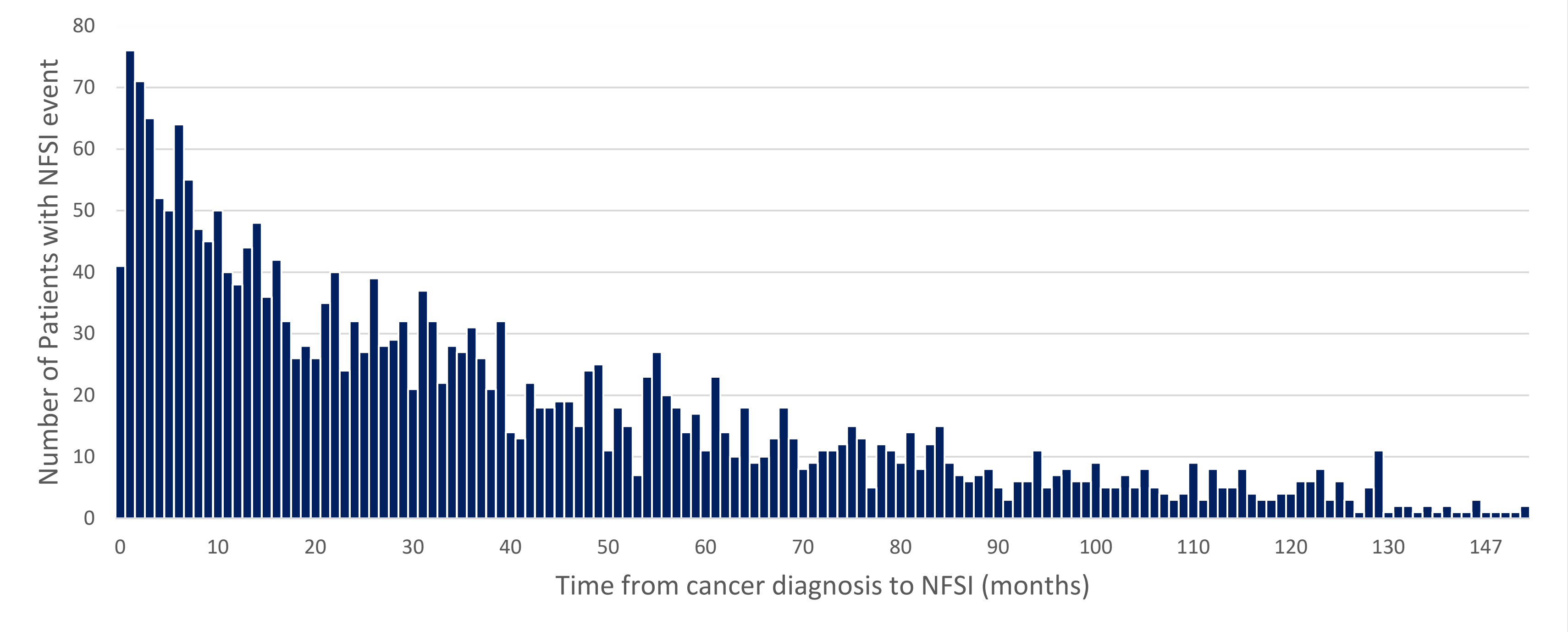

eFigure 2. Distribution of Time From Date of Cancer Diagnosis to Nonfatal Self-injury (NFSI) Event

© 2021 Noel CW et al. *JAMA Network Open.*
