# Supplementary material for: Incidence of and Factors Associated With Nonfatal Self-injury After a Cancer Diagnosis in Ontario, Canada
Source: JAMA Netw Open. 2021 Sep 24;4(9):e2126822. doi: 10.1001/jamanetworkopen.2021.26822 (PMC8463938; doi:10.1001/jamanetworkopen.2021.26822)
Supplement: Supplement 2. — Enhanced Supportive Psycho-oncology Canadian Care (ESPOC) Group Members [file jamanetwopen-e2126822-s002.pdf]

Supplemental Online Content: Nonauthor Collaborators

*Indicates required information. Only first name, last name, and suffix will appear in PubMed.

***Group Name(s): Enhanced Supportive Psycho-oncology Canadian Care (ESPOC) Group**

***First Name and Middle Initial(s)**	***Last Name**	***Suffix (eg, Jr, III)**	Academic Degrees	Institution	Location (city, state/province, country)	Role or Contribution, eg, chair, principal investigator	Group (if more than 1 Group listed in the byline) and/or Subgroup (eg, Steering Committee)
Christopher W	Noel		MD	Univeristy of Toronto	Toronto, ON, Canada		
Antoine	Eskander		MD ScM	Univeristy of Toronto	Toronto, ON, Canada		
Rinku	Sutradhar		PhD	Univeristy of Toronto	Toronto, ON, Canada		
Alyson	Mahar		PhD	Univeristy of Manitoba	Winnipeg, MB, Canada		
Simone N	Vigod		MD MSc	Univeristy of Toronto	Toronto, ON, Canada		
Elie	Isenberg-Grzeda		MDCM	Univeristy of Toronto	Toronto, ON, Canada		
James	Bolton		MD	Univeristy of Manitoba	Winnipeg, MB, Canada		
Wing	Chan		MPH	ICES	Toronto, ON, Canada		
Julie	Deleemans			University of Calgary	Calgary, AB, Canada		
Ravleen	Vasdev		BSc	Univeristy of Toronto	Toronto, ON, Canada		
Victora	Zuk		MSc	Univeristy of Toronto	Toronto, ON, Canada		
Barbara	Haas		MD PhD	Univeristy of Toronto	Toronto, ON, Canada		
Stephanie	Mason		MD PhD	Univeristy of Toronto	Toronto, ON, Canada		
Natalie G	Coburn		MD MPH	Univeristy of Toronto	Toronto, ON, Canada		
Julie	Hallet		MD MSc	Univeristy of Toronto	Toronto, ON, Canada		
